# The Isolation of Discrete Au Nanocrystal/DNA Conjugates and a Personal Journey

**DOI:** 10.1021/acs.nanolett.5c04912

**Published:** 2025-11-19

**Authors:** Daniela Zanchet

**Affiliations:** Institute of Chemistry, Universidade Estadual de Campinas, UNICAMP, Campinas, SP 13083-970, Brazil

The privilege to publish in *Nano Letters* early in my career opened many doors, for which I am proud and grateful.

When *Nano Letters* was launched 25 years ago, nanoscience and nanotechnology were undergoing rapid development, comprising a range of disciplines, such as chemistry, physics, biology, and materials science. The advances in synthetic methods, achieving control over size, morphology, and atomic ordering of nanoparticles, as well as the development and accessibility of advanced tools and techniques for nanoscale characterization and manipulation, were impressive. At that time, I was a first-year postdoctoral fellow at UC Berkeley, California, working in Prof. Alivisatos’s group on the fascinating emerging nano–bio world. I had previously completed my Ph.D. in physics at the Universidade Estadual de Campinas (UNICAMP), São Paulo, Brazil, under the supervision of Dr. Daniel Ugarte. My Ph.D. research focused on the structural properties and self-assembly of Au nanoparticles, conducted at the Brazilian Synchrotron Light Laboratory (LNLS), São Paulo, Brazil. My postdoctoral project aimed to explore the inherent programmability of DNA molecules to create different arrays of nanocrystals, complementing other ongoing projects in the group, and following the pioneering work by Alivisatos’s,^1–3^ Mirkin’s,^4–6^ and other groups. I recall how fascinating the possibility of exploring the recognition properties of biomolecule–nanocrystal conjugates was, both in terms of spatial patterning of nanocrystals and utilizing the properties of nanocrystals to detect biorelated processes.

Our paper, published in the inaugural issue of *Nano Letters*,^7^ addressed the isolation of discrete Au nanocrystal/DNA conjugates that could serve as building blocks with a different number of connecting units. For that, we explored the well-known technique to isolate biomolecules, gel electrophoresis. We successfully separated Au nanocrystal/DNA conjugates formed by a Au nanoparticle of 5 or 10 nm in diameter, linked to 1 to 5 single-stranded DNA molecules with different lengths. We demonstrated that changes in nanocrystal mobility resulting from the presence of an attached DNA molecule could be exploited to isolate building blocks with well-defined numbers of linkages. This strategy was later used to form small groupings of nanocrystals, and the precise control of their formation was explored in other applications.^8,9^ In 25 years, it is remarkable to witness how much the nano–bio theme has expanded and matured, as highlighted in the recent editorial of *Nano Letters*, which addresses the roadmap for the next 25 years and the exciting future ahead.^10^ As for our contribution, it remains one of my most cited papers and is still referenced when discussing the historical evolution of the field.

In the welcome editorial to *Nano Letters*,^11^ Alivisatos emphasized both the vast promise of nanoscience for developing functional materials and the need for patience and persistence in overcoming the challenges ahead. A dedicated forum to present and discuss the results would significantly contribute to its success. The role and prestigious achievements of *Nano Letters* over the years, along with complementary journals that followed, including *ACS Nano*, *ACS Applied Nano Materials*, and *ACS Nanoscience Au*, have confirmed the visionary initiative supported by ACS.

The privilege to publish in *Nano Letters* early in my career opened many doors, for which I am proud and grateful. Back in Brazil in the middle of 2001, I worked as a team leader at the LNLS and as one of the researchers in charge of the nanoscience and nanotechnology program. Other opportunities followed, including projects with industry to address practical problems in heterogeneous catalysis. It was through these applied projects that we were able to build the infrastructure for in situ characterization of catalysts at the synchrotron beamlines at LNLS. The intrinsic connection between fields, in this case, nanomaterials and metal-supported catalysts, similar to what I experienced during my postdoctoral work at the nano–bio interface, became clear, and a new phase in my career began. Nowadays, as Deputy Director of the Institute of Chemistry at UNICAMP, one of the top universities in Latin America, it is inspiring to see many talented research groups working on nanomaterials in our community.

*Nano Letters* has consolidated as one of the most prestigious journals in nanoscience and nanotechnology, and a publication in it is followed with pride and recognition. Having the opportunity to contribute to its inaugural issue was a privilege for a few, yet *Nano Letters*’ reputation is a consequence of the amazing work of many who contributed to the advances in the field. I am thankful for being part of this successful history and congratulate the *Nano Letters* community on this significant milestone.
